# Reporting and Handling of Indeterminate Bone Scan Results in the Staging of Prostate Cancer: A Systematic Review

**DOI:** 10.3390/diagnostics8010009

**Published:** 2018-01-16

**Authors:** Lars J. Petersen, Jesper Strandberg, Louise Stenholt, Martin B. Johansen, Helle D. Zacho

**Affiliations:** 1 Department of Nuclear Medicine, Clinical Cancer Research Centre, Aalborg University Hospital, DK-9000 Aalborg, Denmark; j.strandberg@rn.dk (J.S.); h-zacho@rn.dk (H.D.Z.); 2 Department of Clinical Medicine, Aalborg University, DK-9100 Aalborg, Denmark; 3 The Medical Library, Aalborg University Hospital, DK-9000 Aalborg, Denmark; l.stenholt@rn.dk; 4 Unit of Clinical Biostatistics, Aalborg University Hospital, DK-9000 Aalborg, Denmark; martin.johansen@rn.dk

**Keywords:** bone neoplasms, classification, diagnosis, prostatic neoplasms, radionuclide imaging

## Abstract

Bone scintigraphy is key in imaging skeletal metastases in newly diagnosed prostate cancer. Unfortunately, a notable proportion of scans are not readily classified as positive or negative but deemed indeterminate. The extent of reporting of indeterminate bone scans and how such scans are handled in clinical trials are not known. A systematic review was conducted using electronic databases up to October 2016. The main outcome of interest was the reporting of indeterminate bone scans, analyses of how such scans were managed, and exploratory analyses of the association of study characteristics and the reporting of indeterminate bone scan results. Seventy-four eligible clinical trials were identified. The trials were mostly retrospective (85%), observational (95%), large trials (median 195 patients) from five continents published over four decades. The majority of studies had university affiliation (72%), and an author with imaging background (685). Forty-five studies (61%) reported an indeterminate option for the bone scan and 23 studies reported the proportion of indeterminate scans (median 11.4%). Most trials (44/45, 98%) reported how to handle indeterminate scans. Most trials (*n* = 39) used add-on supplementary imaging, follow-up bone scans, or both. Exploratory analyses showed a significant association of reporting of indeterminate results and number of patients in the study (*p* = 0.024) but failed to reach statistical significance with other variables tested. Indeterminate bone scan for staging of prostate cancer was insufficiently reported in clinical trials. In the case of indeterminate scans, most studies provided adequate measures to obtain the final status of the patients.

## 1. Introduction

Bone scintigraphy (BS) has been the method of choice for staging of skeletal metastases in newly diagnosed prostate cancer for decades, and planar BS continues to be the recommended method across all clinical urological guidelines [1,2,3]. The outcome of BS may in many cases determine the treatment decision of the patients. 

The conclusion from a BS may not always be definitive. In large studies, the proportion of indeterminate scans can amount to 16–26% [4,5]. Bone scans are not specific for metastasis but reflect bone remodeling of any cause. Thus, an imaging avid lesion may not per se represent skeletal metastasis. The diagnostic characteristics of planar BS show sensitivity and specificity of approximately 85% and 75–80% [6,7,8]. 

When clinicians receive imaging results for their patients, they must rely on interpretation by imaging experts. A notable proportion of indeterminate BS results have been reported in large studies with consecutive recruitment [4,5]. The reporting of indeterminate scan results in clinical trials in general remains unclear. However, indeterminate cases are rarely reported in diagnostic test accuracy studies [9]. In clinical practice, handling of patients with indeterminate imaging results can vary widely. Some clinicians may decide to do supplementary imaging on all patients with inconclusive results; others do so very infrequently [4,10]. The reporting of indeterminate imaging findings, and how to deal with them, has not previously been described in cancer imaging.

The purpose of this systematic review was to analyze the extent of reporting of indeterminate BS results in the staging of newly diagnosed prostate cancer, and to explore the extent and methods of supplementary or follow-up imaging to reach a final conclusion. Finally, we tried to identify if any study characteristics were associated with reporting of indeterminate BS results.

## 2. Materials and Methods

### 2.1. Literature Search Strategy

A comprehensive literature search was performed using four different bibliographic databases, MEDLINE (Ovid Technologies, New York, NY, USA), Embase (Ovid Technologies, New York, NY, USA), Web of Science (Clarivate Analytics, Philadelphia, PA, USA), and The Cochrane Library (http://www.cochranelibrary.com). The search period span from the start of each database until 6 October 2016. The search was customized for each database using both controlled thesaurus terms and natural language terms for synonyms (Supplementary material, Table S1). All original references were imported into the reference managing tool RefWorks (Proquest, Ann Arbor, MI, USA) where duplicate references were manually deleted. The references were then imported into the screening and data extraction software Covidence and were evaluated for inclusion in the review.

### 2.2. Eligibility Criteria

According to the PICOS concept (patient, intervention, comparator, outcome, study type), the following eligibility criteria were used. (1) Prostate cancer patients; (2) stage: newly diagnosed; (3) the use of planar bone scintigraphy for the detection of skeletal metastases; (4) no requirements for any comparator; (5) reporting of the original bone scan results (i.e., no registry trials); (6) any study design; (7) a minimum of 20 patients per study. In papers with mixed types of cancers, settings, imaging methods, etc., data should be extractable in accordance with the seven eligibility criteria. At first, all papers were reviewed for eligibility by reading the title and abstract. Papers not rejected based on title/abstract were retrieved for full text reading. Two independent readers performed the selection and subsequent extraction process. The protocol for this review was not registered in a public database. The systematic review was performed in accordance with the Preferred Reporting Items for Systematic Reviews and Meta-Analysis (PRISMA) guideline [11]. 

### 2.3. Reporting of Bone Scan Results

Each paper was reviewed for the reporting of bone scan results (dichotomous outcome versus non-dichotomous outcome). Papers with non-dichotomous outcome were classified by their way of reporting the bone scan and any use of additional imaging (e.g., computed tomography or magnetic resonance imaging) used to clarify indeterminate bone scan results.

### 2.4. Epidemiological and Methodological Analyses of the Eligible Studies

We extracted information about study design (interventional or non-interventional), patient enrollment (prospective versus retrospective), selection of patients (consecutive or non-consecutive enrolment), number of included patients, affiliation to university or university hospital, affiliation to an imaging department, number of authors, geographical region, publication year, journal name, impact factor (Thomson Reuters, 2016 if not mentioned otherwise), indexation in MEDLINE, and research domain. The assessment of trial methodology was based on the actual reporting in the original papers. This implied that an item was classified as absent is not specifically reported in the paper. By example, a trial was classified as a prospective trial only if the word ‘prospective’ was mentioned, the terminology was clear (e.g., “we enrolled”) or the trial was classified as an interventional trial. All trials were classified as observational unless the trial was reported as experimental or interventional, or it was a randomized or diagnostic test accuracy study (cross-sectional cohort) with appropriate ethical approval. Finally, unbiased recruitment of patients was acknowledged only if the phrase “consecutive” or “unselected” was used or it was clear that a trial included all patients or an unbiased selection of patients examined in a well-defined period and the eligibility criteria were specified. In the case of diverging options, e.g., a study included both retrospective and prospectively recruited patients [12] the largest sample determined the study methodology classification. We did not look for duplicate reporting of data even though some trials appear to use data from the same population for separate purposes [13,14]. 

### 2.5. Statistics

Descriptive statistics included calculation of median and range. Fisher’s exact test was used for analysis of reporting or not of indeterminate bone scan results with all variables. In cases of a suspect trend, e.g., number of authors, year of publication, and impact factor, we used logistic regression. 

### 2.6. Approvals

The study did not contain individual data but summary data from previously published papers. There are no requirements for ethical approval or informed consent according to national legislation. 

## 3. Results

### 3.1. Literature Search and Study Demographics

**Figure 1:**
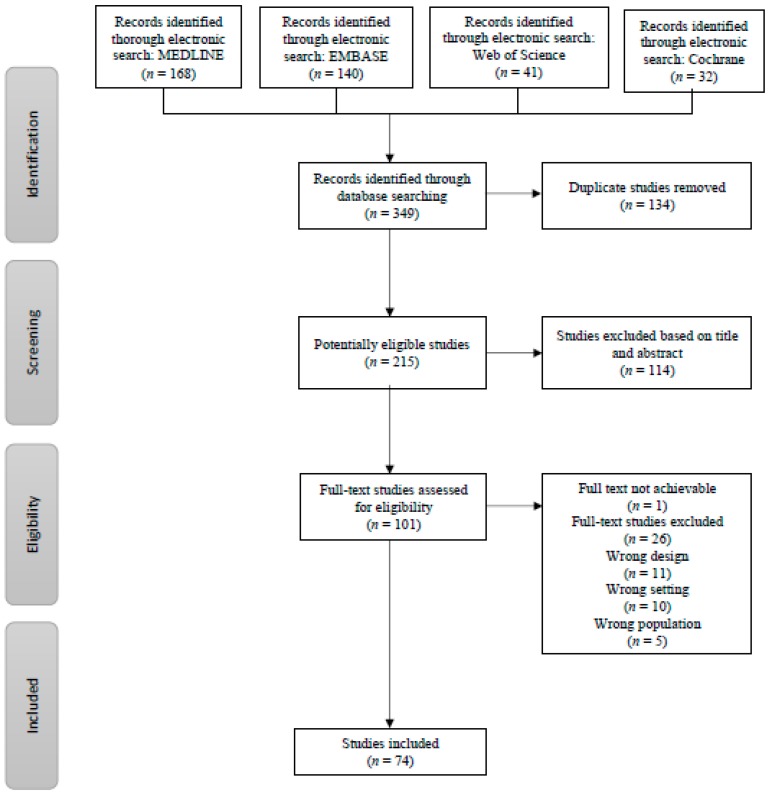
Flow chart of the search and selection process.

The systematic literature search identified 349 individual publications from four databases, which was reduced to 215 papers after removal of duplicates (Figure 1). A total of 114 papers were rejected based on title and abstract, and 101 reports were available for full-text reading. One report was not achievable in full text, 26 papers were found ineligible, thus resulting in 74 papers for data extraction (Figure 1) (Supplementary material, Table S2). The study demographics showed a span of four decades of research, with a median year of publication of 2003; most papers were published in urological papers followed by imaging and oncology journals (Table 1). Half of the papers originated from Europe, but the regional distribution covered five continents. The vast majority of papers originated from university departments, and had at least one author with affiliation to an imaging department. The majority of the trials were retrospective and observational; unbiased recruitment was ensured on approximately 60 percent of the trials. 

### 3.2. Reporting of Indeterminate Bone Scan Results

Forty-five (60.8%) of the papers reported an option for the BS to be inconclusive. A three-level disease classification (positive, negative, indeterminate) was used in 40 trials, where five trials classified the BS results on a scale from four to seven options. Twenty-three of the 45 papers reported the number of patients with indeterminate scan results. The median proportion of such results was 11.4% (range 0.2–28.5%). The vast majority of these studies reported how they handled indeterminate scan results (44 of 45 studies, Table 2). Most trials (*n* = 39) used add-on supplementary imaging; follow up bone scans, or both, whereas three studies declared indeterminate BS as negative for skeletal metastasis, and one study used a consensus reading of the BS by multiple readers. One diagnostic test accuracy study handled indeterminate bone scans as negative and positive (sensitivity analysis) for calculation of diagnostic characteristics of BS [15]. The supplementary imaging was computed tomography (CT) or magnetic resonance imaging (MRI) in most cases, whereas X-ray was used alone or in combination with CT/MRI in 13 studies. A few studies used single photon emission computed tomography (SPECT), SPECT/CT or did not specify the applied methodologies. The results of supplementary follow-up imaging are seldom reported and thus not part of the scope of this paper.

Twenty-nine (29/74, 39.2%) of the papers reported the BS reading solely with a dichotomous outcome as negative or positive for skeletal metastases. In five cases, the authors stated that positive and/or negative BS results were confirmed by supplementary imaging, either for all cases [16,17,18] or selected cases [19,20]. 

### 3.3. Association of Research Methodology and BS Reporting

**Figure 2:**
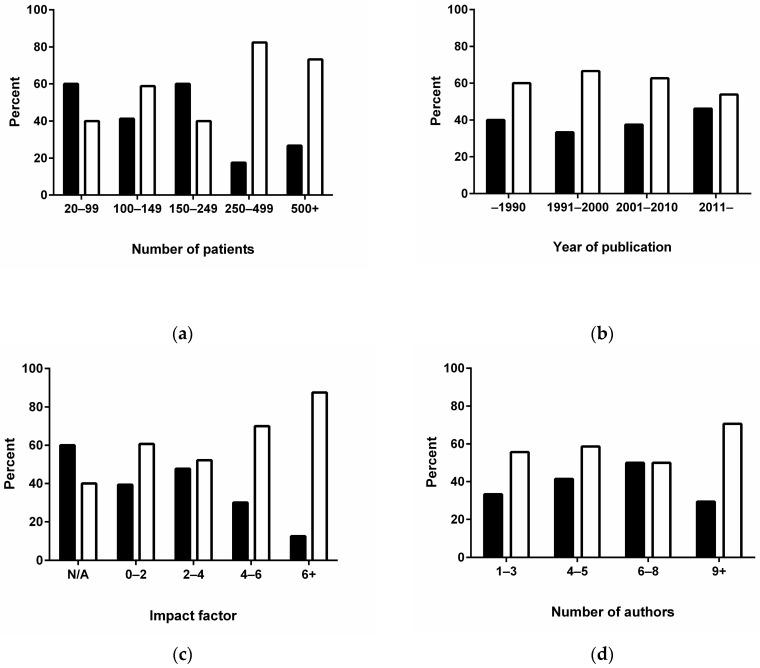
The association of variables with reporting of equivocal bone scans. There was a statistically significant trend with the number of patients in the trials with number of patients in each trial (**a**); but not with the year of publication (**b**); the impact factor of the journals (**c**) or the number of authors (**d**). Reporting of equivocal is shown with open bars, no equivocal scans with closed bars.

We performed exploratory statistical analysis to analyze if certain study characteristics were associated with reporting of indeterminate bone scan results. The results of variables on an ordinal scale showed a statistically significant trend for reporting of indeterminate results with the number of patients in the study (*p* = 0.024, Figure 2a), but not significant differences for year of publication (*p* = 0.423, Figure 2b), impact factor (0.686, Figure 2c), or number of authors (*p* = 0.835, Figure 2d). None of the dichotomous variables showed any significant differences (Table 3). Numerically assesses, reporting of indeterminate BS results was observed in journals with high impact factor (Figure 2c), whereas lack of reporting of indeterminate results was apparent in oncology journals, reports from Asia, oncology journals, and studies with non-consecutive patients (Table 3). There were no apparent differences among papers with or without university affiliation, imaging affiliation, study design, and MEDLINE indexation.

## 4. Discussion

Clinicians require definite answers from imaging of their patients, but some lesions are difficult to interpret. Setting a stage where imaging results can only be classified as positive or negative does not represent the clinical reality in imaging. This has been documented with BS in large clinical trials [4,5]. How this dilemma is solved in clinical practice remains to be documented. This paper described, to the best of our knowledge, the first public available review of the reporting and handling of indeterminate bone scan results among a very large sample of clinical trials. The study showed a lack of methodological rigor for proper classification of the uncertainty with bone scan results in a large proportion of the studies.

The reporting of indeterminate trials in oncology has not been described. However, in diagnostic test accuracy trials in general, the issue is well established. A recent report identified 1156 original diagnostic papers in 22 systematic reviews and showed reporting of uninterpretable, indeterminate, and missing results in only 35% of the reports [9]. The findings by Shinkins et al. [9] and the present data are somewhat compatible; even though approximately 60% of the trials reported an indeterminate option for BS, only 23 studies (31%) actually showed data for indeterminate BS results. Still, we mainly aimed to identify indeterminate options for the bone scan; we did not look for uninterpretable or missing data. Finally, we are well aware that clinical trials, published in clinical journals, as with most of the references presented in this systematic review, are different in research methodology from diagnostic test accuracy studies, with the primary focus on the validity of the index test. However, methodological errors in the reporting of indeterminate trial results are present in both diagnostic test accuracy trials and clinical trials where the reference test is a dichotomous outcome, bone metastases present or absent. 

Planar bone scans are widely recommended across urological guidelines for the staging of newly diagnosed prostate cancer [1,2,3]. Technical developments in form of single photon emission tomography/computer tomography (SPECT/CT) have shown to improve specificity over planar bone scans [21,22]. Imaging experts may argue that planar BS is obsolete in the presence of SPECT/CT. However, urological guidelines do not see it that way. Except for the latest version of the prostate guideline from National Comprehensive Cancer Network, no clinical guidelines even mention SPECT/CT. In addition, whole body SPECT/CT is often used as an add-on to indeterminate planar bone scans, not as the method of choice per se [10,21,23]. A multitude of imaging modalities has evolved, including positron emission tomography (PET)/CT with various tracers and diffusion-weighted magnetic resonance imaging (DW-MRI). There are no overviews of the reporting of indeterminate of scans on a patient or lesion level with these methodologies. Indeterminate results are seldom mentioned in diagnostic trials for bone metastases with PET/CT in prostate, but when it occurred, indeterminate lesions may occur in more than 17% of the cases [24]. 

The vast majority of studies reporting on indeterminate bone scan results presented data for the attempts to get a final diagnosis. Most studies used supplementary anatomical imaging, e.g., targeted X-ray, CT, and/or MRI. The validity and complexity of the examinations, which follow the BS, to obtain a clinical relevant bone status were not examined in detail here. This follow = up is clinical relevant for individual patients, but of minor importance for the validity of the BS as an index test in staging of patients in general. 

In an attempt to identify reports with proper reporting of unclear imaging findings, we performed analyses of study characteristics. Besides the size of the trial, no variable was statistically associated with such reporting. Reporting of indeterminate results was observed in journals with a high impact factor, whereas lack of reporting of indeterminate results was apparent in oncology journals, reports from Asia, and studies with non-consecutive patients.

The median proportion of indeterminate scan results, among those 23 papers reporting such details, was 11%. This figure is notably lower than the 16–26% reported previously [4,5]. It should, however, be noted, that a large proportion of the included studies were retrospective trials, and enrollment of consecutive patients was noted in only approximately 60% of the trials. The results remain speculative if patients with inconclusive imaging findings have been removed from the study population. Such considerations are highly relevant for societies and authorities that use clinical trial results for guidelines and treatment algorithms. Strict research methodology is usually confined to randomized controlled trials and diagnostic test accuracy studies, e.g., The Standards for Reporting of Diagnostic Accuracy Studies (STARD), which describe the reporting criteria for diagnostic test accuracy studies [25]. The initial STARD checklist from 2003 required disclosure of how indeterminate (as well as missing) index tests were handled. 

This study comprised studies with BS. Still, the underlying research methodological issue of how to handle unclear study results is general. Unclear imaging findings are not restricted to bone scans; there are a plenitude of other examples, e.g., in renal and adrenal mass imaging [26,27], pulmonary perfusion imaging [28], and brain imaging [29]. The topic of missing or indeterminate results of diagnostic testing has been debated for a long time in other areas of medicine besides imaging [30,31,32].

Even though planar bone scans may be replaced by more accurate imaging methods, indeterminate imaging results may still occur. Imaging experts as well as clinicians may be aware of the situation that some investigations may be inconclusive. Based on pre-existing risk factors and the localization and extent of lesions, the imaging expert should guide the clinicians on the requirement for any supplementary imaging, including the choice of appropriate methods. Complicated cases may be discussed at multi-disciplinary team conferences. Such a recommendation is valid for planar bone scans as well as other modalities.

## 5. Conclusions

Reporting and analysis of indeterminate imaging results were inadequately handled in staging for bone metastases in prostate cancer. We encourage imaging experts to abstain from a dichotomous classification if such is not obvious. In any imaging study, the authors should adhere to, and editors require compliance with, relevant sections of the STARD recommendations for reporting of diagnostic studies.

## Figures and Tables

**Table 1:** Study demographics of the 74 included papers.

Variable	Data
Reporting of equivocal BS results, *n* (%)	
Yes	45 (60.8%)
No	29 (39.2%)
Year of publication, median (range)	2003 (1974–2016)
Number of patients, median (range)	195 (25–1515)
Number of authors, median (range)	5 (1–14)
Study design	
Experimental	4 (5.4%)
Observational	70 (94.6%)
Patient enrollment	
Prospective	11 (14.9%)
Retrospective	63 (85.1%)
Consecutive patients	
Yes	45 (60.8%)
No	29 (39.2%)
Research domain, *n* (%)	
Urology	36 (48.6%)
Imaging	18 (24.3%)
Oncology	16 (21.6%)
Other	4 (5.5%)
Geographical origin, *n* (%)	
Europe	37 (50.0%)
Asia	22 (29.7%)
North America	11 (14.9%)
Middle East	3 (4.1%)
Africa	1 (1.4%)
University affiliation, *n* (%)	
Yes	53 (71.6%)
No	21 (28.4%)
Imaging affiliation, *n* (%)	
Yes	50 (67.6%)
No	24 (32.4%)
Impact factor, *n* (%)	
Journals without impact factor	5 (6.8%)
Journals with impact factor	69 (93.2%)
Impact factor, median (range)	2.309 (0.815–33.405)
MEDLINE indexation, *n* (%)	
Yes	70 (94.6%)
No	4 (5.4)

Abbreviations: BS, bone scintigraphy.

**Table 2:** Handling of equivocal bone scan results.

Variable	Data
Reporting of equivocal BS results, *n*	45
Described handling of equivocal results, *n* (%)	
Yes	44 (97.8%)
No	1 (2.2%)
Supplementary imaging only, *n*	36
Type of supplementary imaging and/or management	
X-ray	9
CT or MRI	21
X-ray and/or CT/MRI	4
Other	2
Supplementary imaging and follow up bone scans, *n*	2
Follow up bone scans only, *n*	1
Equivocal bone scans considered negative for skeletal metastases, *n*	3
Sensitivity analysis, *n*	1
Third party arbitrator of equivocal bone scans, *n*	1

Abbreviations: BS, bone scintigraphy; CT, computed tomography, MRI, magnetic resonance imaging.

**Table 3:** Study design and reporting of equivocal scan results.

Variable	Numbers	Eq Not Reported (%)	Eq Reported (%)	*p*-Value
Geographical origin				0.128
Europe	37	35.1	64.9	
Asia	22	59.1	40.9	
North America	11	18.2	81.8	
Middle East	3	33.3	66.7	
Africa	1	0.0	100.0	
Research domain				0.190
Urology	36	33.3	66.7	
Imaging	18	33.3	66.7	
Oncology	16	62.5	37.5	
Other	4	25.6	75.0	
University affiliation, *n* (%)				0.603
Yes	53	41.5	58.5	
No	21	33.3	66.7	
Imaging affiliation, *n* (%)				0.803
Yes	50	38.0	62.0	
No	24	41.7	58.3	
Study design				0.642
Experimental	4	50.0	50.0	
Observational	70	38.6	61.4	
Patient enrollment				1.000
Prospective	11	36.4	63.6	
Retrospective	63	39.7	60.3	
Consecutive patients				0.092
Yes	45	31.1	68.9	
No	29	51.7	48.3	
MEDLINE indexation				0.642
Yes	70	38.6	61.4	
No	4	50.0	50.0	

## Supplementary Materials

The following are available online at www.mdpi.com/2075-4418/08/1/9/s1.
