# Supplementary material for: Reporting and Handling of Indeterminate Bone Scan Results in the Staging of Prostate Cancer: A Systematic Review
Source: Diagnostics (Basel). 2018 Jan 16;8(1):9. doi: 10.3390/diagnostics8010009 (PMC5871992; doi:10.3390/diagnostics8010009)
Supplement: Supplementary file 1 [file diagnostics-08-00009-s001.pdf]

Table S1

Search strategy Equivocal 6th of October 2016

Search results EMBASE

1	exp prostate cancer/	169710
2	((prostat* gland cancer or prostat* cancer* or prostat* adenocarcinoma* or prostat* carcinoma* or prostat* intraepithelial neoplas* or prostat* duct carcinoma* or prostat* neoplas*) not recurrent not relapse).ti,ab,ot.	139190
3	1 or 2	189085
4	bone metastasis/	34721
5	(bone invasion or bone metasta* or metasta* bone or ilium metasta* or osseous metasta* or osteoblast* metasta* or osteoplast* metasta* or osteoclast* metasta* or skelet* metasta* or skull metasta*).ti,ab,ot.	25765
6	4 or 5	40918
7	exp bone scintiscanning/	25497
8	(bone scan* or bone scintigr* or bone scintimetr* or bone scintiscan* or bone tumo?r scan* or oteoscintigr* or skelet* scintigr* or skelet* scan* or skelet* scintiscan* or skelet* scintimetr*).ti,ab,ot.	17392
9	technetium 99m/	34486
10	(99m Tc or 99mTc or colloid* technetium or technetium 99* or Tc99* or Tc 99*).ti,ab,ot.	51099
11	7 or 8 or 9 or 10	86131
12	(newly diagnos* or staging).ti,ab,ot.	150003
13	3 and 6 and 11 and 12	322
14	limit 13 to english language	288
15	limit 14 to article	140

Search results MEDLINE

1	exp prostate cancer/	107922
2	((prostat* gland cancer or prostat* cancer* or prostat* adenocarcinoma* or prostat* carcinoma* or prostat* intraepithelial neoplas* or prostat* duct carcinoma* or prostat* neoplas*) not recurrent not relapse).ti,ab,ot.	102377
3	1 or 2	122603
4	(bone neoplasms/ and neoplasm metastasis/) or Bone Neoplasms/sc	21147
5	(bone invasion or bone metasta* or metasta* bone or ilium metasta* or osseous metasta* or osteoblast* metasta* or osteoplast* metasta* or osteoclast* metasta* or skelet* metasta* or skull metasta*).ti,ab,ot.	29399
6	4 or 5	12572

7	(bone scan* or bone scintigr* or bone scintimetr* or bone scintiscan* or bone tumo?r scan* or oteoscintigr* or skelet* scintigr* or skelet* scan* or skelet* scintiscan* or skelet* scintimetr*).ti,ab,ot.	12062
8	technetium 99m/	19616
9	(99m Tc or 99mTc or colloid* technetium or technetium 99* or Tc99* or Tc 99*).ti,ab,ot.	37480
10	7 or 8 or 9	56577
11	(newly diagnos* or staging).ti,ab,ot.	97523
12	3 and 6 and 10 and 11	193
13	limit 12 to english language	171
14	limit 13 to journal article	168

Search Results Web of Science

1	TS=((prostat* cancer* or prostat* adenocarcinoma* or prostat* carcinoma* or prostat* intraepithelial neoplas* or prostat* neoplas*) not recurrent not relapse)	183629
2	TS=(bone invasion or bone metasta* or ilium metasta* or osseous metasta* or osteoblast* metasta* or osteoplast* metasta* or osteoclast* metasta* or skelet* metasta* or skull metasta*)	44767
3	TS=(bone scan* or bone scintigr* or bone scintimetr* or bone scintiscan* or bone tumor* scan* or bone tumour* scan* or oteoscintigr* or skelet* scintigr* or skelet* scan* or skelet* scintiscan* or skelet* scintimetr*)	49162
4	TS=(99m Tc or 99mTc or colloid* technetium or technetium 99 or Tc99 or Tc 99 or technetium 99m or Tc99m)	46837
5	TS=(newly diagnos* or staging)	1337405
6	#5 AND #4 AND #3 AND #2 AND #1	50
7	(#6) AND LANGUAGE: (English)	47
8	(#7) AND LANGUAGE: (English) AND DOCUMENT TYPES: (Article)	41

Table S2. list of included papers listed in alphabetic order [1-74]

Reference List

1. Ahel, M.Z.; Kovacic, K.; Tarle, M. Cross-correlation of serum chromogranin a, %-f-psa and bone scans in prostate cancer diagnosis. *Anticancer research* **2001**, *21*, 1363-1366.
2. Al-Ghazo, M.; Ghalayini, I.F.; Al-Azab, R.; Bani-Hani, I.; Barham, A.; Haddad, Y. Do all patients with newly diagnosed prostate cancer need staging radionuclide bone scan? A retrospective study. *International braz j urol : official journal of the Brazilian Society of Urology* **2010**, *36*, 685-682.
3. Ayyathurai, R.; Mahapatra, R.; Rajasundaram, R.; Srinivasan, V.; Archard, N.P.; Toussi, H. A study on staging bone scans in newly diagnosed prostate cancer. *Urologia internationalis* **2006**, *76*, 209-212.
4. Boughattas, S.; Letaief, B.; Hassine, H.; Chatti, K.; Essabah, H. Bone scan in initial staging of prostate cancer. *La Tunisie medicale* **2003**, *81*, 400-406.

5. Briganti, A.; Passoni, N.; Ferrari, M.; Capitanio, U.; Suardi, N.; Gallina, A.; Da Pozzo, L.F.; Picchio, M.; Di Girolamo, V.; Salonia, A., *et al.* When to perform bone scan in patients with newly diagnosed prostate cancer: External validation of the currently available guidelines and proposal of a novel risk stratification tool. *Eur Urol* **2010**, *57*, 551-558.
6. Bruwer, G.; Heyns, C.F.; Allen, F.J. Influence of local tumour stage and grade on reliability of serum prostate-specific antigen in predicting skeletal metastases in patients with adenocarcinoma of the prostate. *European urology* **1999**, *35*, 223-227.
7. Chien, T.-M.; Lu, Y.-M.; Geng, J.-H.; Huang, T.-Y.; Ke, H.-L.; Huang, C.-N.; Li, C.-C.; Chou, Y.-H.; Wu, W.-J.; Huang, S.-P. Predictors of positive bone metastasis in newly diagnosed prostate cancer patients. *Asian Pacific journal of cancer prevention : APJCP* **2016**, *17*, 1187-1191.
8. Chong, A.; Hwang, I.; Ha, J.M.; Yu, S.H.; Hwang, E.C.; Yu, H.S.; Kim, S.O.; Jung, S.I.; Kang, T.W.; Kwon, D.D.*, et al.* Application of bone scans for prostate cancer staging: Which guideline shows better result? *Can Urol Assoc J* **2014**, *8*, E515-519.
9. Chrapko, B.E.; Nocun, A.; Golebiewska, R.; Jankowska, H.; Zaorska-Rajca, J. Bone turnover markers and bone scintigraphy in the evaluation of skeletal metastases. Via Medica (Ul. Swietokrzyska 73, Gdansk 80-180, Poland): Poland, 2005; Vol. 8, pp 100-104.
10. Chybowski, F.M.; Keller, J.J.; Bergstralh, E.J.; Oesterling, J.E. Predicting radionuclide bone scan findings in patients with newly diagnosed, untreated prostate cancer: Prostate specific antigen is superior to all other clinical parameters. *The Journal of urology* **1991**, *145*, 313-318.
11. Corrie, D.; Timmons, J.H.; Bauman, J.M.; Thompson, I.M. Efficacy of follow-up bone scans in carcinoma of the prostate. John Wiley and Sons Inc. (P.O.Box 18667, Newark NJ 07191-8667, United States): United States, 1988; Vol. 61, pp 2453-2454.
12. Damle, N.A.; Bal, C.; Bandopadhyaya, G.P.; Kumar, L.; Kumar, P.; Malhotra, A.; Lata, S. The role of 18f-fluoride pet-ct in the detection of bone metastases in patients with breast, lung and prostate carcinoma: A comparison with fdg pet/ct and 99mtc-mdp bone scan. *Japanese journal of radiology* **2013**, *31*, 262-269.
13. De Nunzio, C.; Leonardo, C.; Franco, G.; Esperto, F.; Brassetti, A.; Simonelli, G.; Dente, D.; De Dominicis, C.; Tubaro, A. When to perform bone scan in patients with newly diagnosed prostate cancer: External validation of a novel risk stratification tool. *World journal of urology* **2013**, *31*, 365-369.
14. Evangelista, L.; Cimitan, M.; Zattoni, F.; Guttilla, A.; Zattoni, F.; Saladini, G. Comparison between conventional imaging (abdominal-pelvic computed tomography and bone scan) and (18)f]choline positron emission tomography/computed tomography imaging for the initial staging of patients with intermediate- tohigh-risk prostate cancer: A retrospective analysis. *Scand J Urol* **2015**, *49*, 345-353.
15. Gerber, G.; Chodak, G.W. Assessment of value of routine bone scans in patients with newly diagnosed prostate cancer. *Urology* **1991**, *37*, 418-422.
16. Gleave, M.E.; Coupland, D.; Drachenberg, D.; Cohen, L.; Kwong, S.; Goldenberg, S.L.; Sullivan, L.D. Ability of serum prostate-specific antigen levels to predict normal bone scans in patients with newly diagnosed prostate cancer. *Urology* **1996**, *47*, 708-712.
17. Goodman, C.M.; Cumming, J.A.; Ritchie, A.W.; Chisholm, G.D. Prognostic value of raised prostatic acid phosphatase and negative skeletal scintigraphy in prostatic cancer. *British journal of urology* **1991**, *67*, 622-625.
18. Haukaas, S.; Roervik, J.; Halvorsen, O.J.; Foelling, M. When is bone scintigraphy necessary in the assessment of newly diagnosed, untreated prostate cancer? *British journal of urology* **1997**, *79*, 770-776.
19. Hayward, S.J.; McIvor, J.; Burdge, A.H.; Jewkes, R.F.; Williams, G. Staging of prostatic carcinoma with radionuclide bone scintigraphy and lymphography. *The British journal of radiology* **1987**, *60*, 79-81.
20. Hirobe, M.; Takahashi, A.; Hisasue, S.-I.; Kitamura, H.; Kunishima, Y.; Masumori, N.; Iwasawa, A.; Fujimori, K.; Hasegawa, T.; Tsukamoto, T. Bone scanning--who needs it among patients with newly diagnosed prostate cancer? *Japanese journal of clinical oncology* **2007**, *37*, 788-792.

21. Ho, C.C.K.; Seong, P.K.; Zainuddin, Z.M.; Abdul Manaf, M.R.; Parameswaran, M.; Razack, A.H.A. Retrospective study of predictors of bone metastasis in prostate cancer cases. *Asian Pacific journal of cancer prevention : APJCP* **2013**, *14*, 3289-3292.
22. Jacobson, A.F. Association of prostate-specific antigen levels and patterns of benign and malignant uptake detected. On bone scintigraphy in patients with newly diagnosed prostate carcinoma. *Nuclear medicine communications* **2000**, *21*, 617-622.
23. Jaukovic, L.; Adjinovic, B.; Cerovic, S.; Joksimovic, M.; Soldatovic, Z. Is bone scintigraphy necessary in initial staging of prostate cancer patients? *Hellenic journal of nuclear medicine* **2011**, *14*, 126-130.
24. Kemp, P.M.; Maguire, G.A.; Bird, N.J. Which patients with prostatic carcinoma require a staging bone scan? *British journal of urology* **1997**, *79*, 611-614.
25. Kikuchi, E.; Nakashima, J.; Ishibashi, M.; Ohigashi, T.; Oya, M.; Nakagawa, K.; Miyajima, A.; Murai, M. Usefulness of alpha1-antichymotrypsin-psa complex for predicting bone metastases of prostate cancer. *Urology* **2006**, *68*, 371-375.
26. Koga, H.; Naito, S.; Koto, S.; Sakamoto, N.; Nakashima, M.; Yamasaki, T.; Noma, H.; Kumazawa, J. Use of bone turnover marker, pyridinoline cross-linked carboxyterminal telopeptide of type i collagen (ictp), in the assessment and monitoring of bone metastasis in prostate cancer. *The Prostate* **1999**, *39*, 1-7.
27. Kosuda, S.; Yoshimura, I.; Aizawa, T.; Koizumi, K.; Akakura, K.; Kuyama, J.; Ichihara, K.; Yonese, J.; Koizumi, M.; Nakashima, J., *et al.* Can initial prostate specific antigen determinations eliminate the need for bone scans in patients with newly diagnosed prostate carcinoma? A multicenter retrospective study in japan. *Cancer* **2002**, *94*, 964-972.
28. Lai, M.H.Y.; Luk, W.H.; Chan, J.C.S. Predicting bone scan findings using spsa in patients newly diagnosed of prostate cancer: Feasibility in asian population. *Urologic oncology* **2011**, *29*, 275-279.
29. Lecouvet, F.E.; El, M.J.; Collette, L.; Coche, E.; Danse, E.; Jamar, F.; Machiels, J.P.; Vande, B.B.; Omoumi, P.; Tombal, B. Can whole-body magnetic resonance imaging with diffusion-weighted imaging replace tc 99m bone scanning and computed tomography for single-step detection of metastases in patients with high-risk prostate cancer? *Eur Urol* **2012**, *62*, 68-75.
30. Lee, N.; Fawaaz, R.; Olsson, C.A.; Benson, M.C.; Petrylak, D.P.; Schiff, P.B.; Bagiella, E.; Singh, A.; Ennis, R.D. Which patients with newly diagnosed prostate cancer need a radionuclide bone scan? An analysis based on 631 patients. Elsevier Inc. (360 Park Avenue South, New York NY 10010, United States): United States, 2000; Vol. 48, pp 1443-1446.
31. Lee, S.H.; Chung, M.S.; Park, K.K.; Yom, C.D.; Lee, D.H.; Chung, B.H. Is it suitable to eliminate bone scan for prostate cancer patients with psa < 20 ng/ml? *World journal of urology* **2012**, *30*, 265-269.
32. Lentle, B.C.; McGowan, D.G.; Dierich, H. Technetium 99m polyphosphate bone scanning in carcinoma of the prostate. 1974; Vol. 46, pp 543-548.
33. Lin, K.; Szabo, Z.; Chin, B.B.; Civelek, A.C. The value of a baseline bone scan in patients with newly diagnosed prostate cancer. *Clinical nuclear medicine* **1999**, *24*, 579-582.
34. Lorente, J.A.; Valenzuela, H.; Morote, J.; Gelabert, A. Serum bone alkaline phosphatase levels enhance the clinical utility of prostate specific antigen in the staging of newly diagnosed prostate cancer patients. *European journal of nuclear medicine* **1999**, *26*, 625-632.
35. Lu, Y.M.; Chien, T.M.; Ke, H.L.; Huang, S.P.; Huang, C.N. The most suitable guidelines for performing bone scans in prostate cancer staging - one southern taiwan medical center's results. Elsevier: Netherlands, 2015; p no pagination.
36. McArthur, C.; McLaughlin, G.; Meddings, R.N. Changing the referral criteria for bone scan in newly diagnosed prostate cancer patients. *The British journal of radiology* **2012**, *85*, 390-394.
37. Merdan, S.; Womble, P.R.; Miller, D.C.; Barnett, C.; Ye, Z.; Linsell, S.M.; Montie, J.E.; Denton, B.T. Toward better use of bone scans among men with early-stage prostate cancer. *Urology* **2014**, *84*, 793-798.

38. Miller, P.D.; Eardley, I.; Kirby, R.S. Prostate specific antigen and bone scan correlation in the staging and monitoring of patients with prostatic cancer. *British journal of urology* **1992**, *70*, 295-298.
39. Morote, J.; Lorente, J.A.; Encabo, G. Prostate carcinoma staging. Clinical utility of bone alkaline phosphatase in addition to prostate specific antigen. *Cancer* **1996**, *78*, 2374-2378.
40. Moslehi, M.; Cheki, M.; Salehi-Marzijarani, M.; Amuchastegui, T.; Gholamrezanezhad, A. Predictors of bone metastasis in pre-treatment staging of asymptomatic treatment-naive patients with prostate cancer. *Revista espanola de medicina nuclear e imagen molecular* **2013**, *32*, 286-289.
41. O'Donoghue, J.M.; Rogers, E.; Grimes, H.; McCarthy, P.; Corcoran, M.; Bredin, H.; Given, H.F. A reappraisal of serial isotope bone scans in prostate cancer. *The British journal of radiology* **1993**, *66*, 672-676.
42. Oesterling, J.E.; Martin, S.K.; Bergstralh, E.J.; Lowe, F.C. The use of prostate-specific antigen in staging patients with newly diagnosed prostate cancer. *JAMA* **1993**, *269*, 57-60.
43. O'Sullivan, J.M.; Norman, A.R.; Cook, G.J.; Fisher, C.; Dearnaley, D.P. Broadening the criteria for avoiding staging bone scans in prostate cancer: A retrospective study of patients at the royal marsden hospital. *BJU international* **2003**, *92*, 685-689.
44. Ozgur, B.C.; Gultekin, S.; Ekici, M.; Yilmazer, D.; Alper, M. A narrowing range of bone scan in newly diagnosed prostate cancer patients: A retrospective comparative study. *Urology annals* **2015**, *7*, 193-198.
45. Palmedo, H.; Marx, C.; Ebert, A.; Kreft, B.; Ko, Y.; Turler, A.; Vorreuther, R.; Gohring, U.; Schild, H.H.; Gerhardt, T., *et al.* Whole-body spect/ct for bone scintigraphy: Diagnostic value and effect on patient management in oncological patients. *Eur J Nucl Med Mol Imaging* **2014**, *41*, 59-67.
46. Pasoglou, V.; Larbi, A.; Collette, L.; Annet, L.; Jamar, F.; Machiels, J.-P.; Michoux, N.; Vande Berg, B.C.; Tombal, B.; Lecouvet, F.E. One-step tnm staging of high-risk prostate cancer using magnetic resonance imaging (mri): Toward an upfront simplified "all-in-one" imaging approach? *The Prostate* **2014**, *74*, 469-477.
47. Paul, R.; Bottermann, R.; Breul, J.; Hartung, R. Isoenzymes of alkaline phosphatase - useful parameters for identification of bone metastasis in prostatic carcinoma? S. Karger AG (Allschwilerstrasse 10, P.O. Box, Basel CH-4009, Switzerland): Germany, 2000; Vol. 23, pp 42-46.
48. Perachino, M.; Di Ciolo, L.; Barbetti, V.; Puppo, P. Procollagen type i carboxyterminal extension peptide in serum: A reliable marker of bone metastatic disease in newly diagnosed prostate cancer? *European urology* **1996**, *29*, 366-369.
49. Pyka, T.; Okamoto, S.; Dahlbender, M.; Tauber, R.; Retz, M.; Heck, M.; Tamaki, N.; Schwaiger, M.; Maurer, T.; Eiber, M. Comparison of bone scintigraphy and 68ga-psma pet for skeletal staging in prostate cancer. *Eur J Nucl Med Mol Imaging* **2016**, *43*, 2114-2121.
50. Rana, A.; Chisholm, G.D.; Christodoulou, S.; McIntyre, M.A.; Elton, R.A. Audit and its impact in the management of early prostatic cancer. *British journal of urology* **1993**, *71*, 721-727.
51. Ritenour, C.W.M.; Abbott, J.T.; Goodman, M.; Alazraki, N.; Marshall, F.F.; Issa, M.M. The utilization of gleason grade as the primary criterion for ordering nuclear bone scan in newly diagnosed prostate cancer patients. *TheScientificWorldJournal* **2009**, *9*, 1040-1045.
52. Rudoni, M.; Antonini, G.; Favro, M.; Baroli, A.; Brambilla, M.; Cardani, G.; Ciardi, L.; Sacchetti, G.M.; Inglese, E. The clinical value of prostate-specific antigen and bone scintigraphy in the staging of patients with newly diagnosed, pathologically proven prostate cancer. *European journal of nuclear medicine* **1995**, *22*, 207-211.
53. Rydh, A.; Tomic, R.; Tavelin, B.; Hietala, S.O.; Damber, J.E. Predictive value of prostate-specific antigen, tumour stage and tumour grade for the outcome of bone scintigraphy in patients with newly diagnosed prostate cancer. *Scandinavian journal of urology and nephrology* **1999**, *33*, 89-93.
54. Sakamoto, K.; Tanaka, F.; Miyazaki, Y.; Ariyoshi, A. Diagnostic procedures for assessment of disease extent in prostatic carcinoma. *The Prostate.Supplement* **1981**, *1*, 47-52.
55. Salonia, A.; Gallina, A.; Camerota, T.C.; Picchio, M.; Freschi, M.; DaPozzo, L.F.; Guazzoni, G.; Fazio, F.; Rigatti, P.; Montorsi, F. Bone metastases are infrequent in patients with newly

diagnosed prostate cancer: Analysis of their clinical and pathologic features. *Urology* **2006**, *68*, 362-366.
56. Sergieva, S.; Kirova, G.; Dudov, A. Current diagnostic approaches in tumor-induced bone disease. *Journal of Buon* **2007**, *12*, 493-504.
57. Spencer, J.A.; Chng, W.J.; Hudson, E.; Boon, A.P.; Whelan, P. Prostate specific antigen level and gleason score in predicting the stage of newly diagnosed prostate cancer. *The British journal of radiology* **1998**, *71*, 1130-1135.
58. Stokkel, M.; Zwinderman, A.; Zwartendijk, J.; Pauwels, E.; van Eck-Smit, B. The value of pretreatment clinical and biochemical parameters in patients with newly diagnosed untreated prostate carcinoma and no indications for bone metastases on the bone scintigram. *European journal of nuclear medicine* **1997**, *24*, 1215-1220.
59. Suzuki, H.; Inoue, Y.; Fujimoto, H.; Yonese, J.; Tanabe, K.; Fukasawa, S.; Inoue, T.; Saito, S.; Ueno, M.; Otaka, A. Diagnostic performance and safety of nmk36 (trans-1-amino-3-18f]fluorocyclobutanecarboxylic acid)-pet/ct in primary prostate cancer: Multicenter phase iib clinical trial. *Japanese journal of clinical oncology* **2016**, *46*, 152-162.
60. Szot, W.; Kostkiewicz, M.; Zajac, J.; Owoc, A.; Bojar, I. Prostate cancer in patients from rural and suburban areas--psa value, gleason score and presence of metastases in bone scan. *Annals of Agricultural and Environmental Medicine : AAEM* **2014**, *21*, 888-892.
61. Tanaka, N.; Fujimoto, K.; Shinkai, T.; Nakai, Y.; Kuwada, M.; Anai, S.; Miyake, M.; Hirayama, A.; Hasegawa, M.; Hirao, Y. Bone scan can be spared in asymptomatic prostate cancer patients with psa of <=20 ng/ml and gleason score of <=6 at the initial stage of diagnosis. *Japanese journal of clinical oncology* **2011**, *41*, 1209-1213.
62. Traill, Z.C.; Talbot, D.; Golding, S.; Gleeson, F.V. Magnetic resonance imaging versus radionuclide scintigraphy in screening for bone metastases. *Clinical radiology* **1999**, *54*, 448-451.
63. Varenhorst, E.; Alund, G.; Lindstrom, E.; Manson, J.C. Bone marrow aspiration biopsy and bone scanning in the staging of prostatic cancer. *British journal of urology* **1983**, *55*, 534-537.
64. Vijayakumar, V.; Vijayakumar, S.; Quadri, S.F.; Blend, M.J. Can prostate-specific antigen levels predict bone scan evidence of metastases in newly diagnosed prostate cancer? *American journal of clinical oncology* **1994**, *17*, 432-436.
65. Wang, Y.; Guo, J.; Xu, L.; Zhao, N.; Xu, Z.; Wang, H.; Zhu, Y.; Jiang, S.; Yang, N.; Yang, Y., *et al.* Should bone scan be performed in chinese prostate cancer patients at the time of diagnosis? *Urologia internationalis* **2013**, *91*, 160-164.
66. Wang, Y.; Wan, F.; Xu, L.; Zhao, N.; Xu, Z.; Wang, H.; Wang, G.; Ye, D.; Guo, J. Is it safe to omit baseline bone scan for newly diagnosed prostate cancer patients? *Urologia internationalis* **2015**, *94*, 342-346.
67. Wolff, J.M.; Bares, R.; Jung, P.K.; Buell, U.; Jakse, G. Prostate-specific antigen as a marker of bone metastasis in patients with prostate cancer. *Urologia internationalis* **1996**, *56*, 169-173.
68. Wolff, J.M.; Zimny, M.; Borchers, H.; Wildberger, J.; Buell, U.; Jakse, G. Is prostate-specific antigen a reliable marker of bone metastasis in patients with newly diagnosed cancer of the prostate? *European urology* **1998**, *33*, 376-381.
69. Wymenga, L.F.; Boomsma, J.H.; Groenier, K.; Piers, D.A.; Mensink, H.J. Routine bone scans in patients with prostate cancer related to serum prostate-specific antigen and alkaline phosphatase. *BJU international* **2001**, *88*, 226-230.
70. Wymenga, L.F.; Groenier, K.; Schuurman, J.; Boomsma, J.H.; Elferink, R.O.; Mensink, H.J. Pretreatment levels of urinary deoxypyridinoline as a potential marker in patients with prostate cancer with or without bone metastasis. *BJU international* **2001**, *88*, 231-235.
71. Yang, G.; Zuo, S.; Ma, C.; Liu, B.; Wang, G.; Wang, X.; Wu, H. The diagnostic correlations of bone scintigraphy, pathological grade and psa for metastatic prostate cancers. *Chinese-German J Clin Oncol* **2009**, *8*, 702-704.
72. Zacho, H.D.; Barsi, T.; Mortensen, J.C.; Mogensen, M.K.; Bertelsen, H.; Josephsen, N.; Petersen, L.J. Prospective multicenter study of bone scintigraphy in consecutive patients with newly diagnosed prostate cancer. *Clin Nucl Med* **2014**, *39*, 26-31.

73. Zaman, M.U.; Fatima, N.; Sajjad, Z. Metastasis on bone scan with low prostate specific antigen (<20 ng/ml) and gleason's score (<8) in newly diagnosed pakistani males with prostate cancer: Should we follow western guidelines? *Asian Pacific journal of cancer prevention : APJCP* **2011**, *12*, 1529-1532.
74. Zaman, M.U.; Fatima, N.; Sajjad, Z.; Hashmi, I.; Khan, K. Higher scrotal uptake ratio of (99m)tc-mdp on bone scans in newly diagnosed prostate cancer: A reliable indicator of pelvic node metastasis. *Annals of Nuclear Medicine* **2012**, *26*, 676-680.
